# Glucose-6-phosphate dehydrogenase mutations in malaria endemic area of Thailand by multiplexed high‐resolution melting curve analysis

**DOI:** 10.1186/s12936-021-03731-0

**Published:** 2021-04-20

**Authors:** Usa Boonyuen, Duantida Songdej, Sasipa Tanyaratsrisakul, Suparat Phuanukoonnon, Kamonwan Chamchoy, Aun Praoparotai, Phonchanan Pakparnich, Sirapapha Sudsumrit, Thomas Edwards, Christopher T. Williams, Rachel L. Byrne, Emily R. Adams, Mallika Imwong

**Affiliations:** 1 Department of Molecular Tropical Medicine and Genetics, Faculty of Tropical Medicine, Mahidol University, Bangkok, 10400 Thailand; 2 Department of Pediatrics, Faculty of Medicine Ramathibodi Hospital, Mahidol University, Bangkok, 10400 Thailand; 3 Asthma & Airway Disease Research Center, University of Arizona, Tucson, AZ 85719 USA; 4 Department of Social and Environmental Medicine, Faculty of Tropical Medicine, Mahidol University, Bangkok, 10400 Thailand; 5 Faculty of Medicine and Public Health, HRH Princess Chulabhorn College of Medical Science, Chulabhorn Royal Academy, Bangkok, 10210 Thailand; 6 Centre for Drugs and Diagnostics, Liverpool School of Tropical Medicine, L3 5QA Liverpool, UK

**Keywords:** G6PD deficiency, G6PD mutations, High-resolution melting, Phenotype, G6PD enzyme activity, G6PD genotype, WST-8

## Abstract

**Background:**

Glucose-6-phosphate dehydrogenase (G6PD) deficiency, the most common enzymopathy in humans, is prevalent in tropical and subtropical areas where malaria is endemic. Anti-malarial drugs, such as primaquine and tafenoquine, can cause haemolysis in G6PD-deficient individuals. Hence, G6PD testing is recommended before radical treatment against vivax malaria. Phenotypic assays have been widely used for screening G6PD deficiency, but in heterozygous females, the random lyonization causes difficulty in interpreting the results. Over 200 G6PD variants have been identified, which form genotypes associated with differences in the degree of G6PD deficiency and vulnerability to haemolysis. This study aimed to assess the frequency of G6PD mutations using a newly developed molecular genotyping test.

**Methods:**

A multiplexed high-resolution melting (HRM) assay was developed to detect eight G6PD mutations, in which four mutations can be tested simultaneously. Validation of the method was performed using 70 G6PD-deficient samples. The test was then applied to screen 725 blood samples from people living along the Thai–Myanmar border. The enzyme activity of these samples was also determined using water-soluble tetrazolium salts (WST-8) assay. Then, the correlation between genotype and enzyme activity was analysed.

**Results:**

The sensitivity of the multiplexed HRM assay for detecting G6PD mutations was 100 % [95 % confidence interval (CI): 94.87–100 %] with specificity of 100 % (95 % CI: 87.66–100 %). The overall prevalence of G6PD deficiency in the studied population as revealed by phenotypic WST-8 assay was 20.55 % (149/725). In contrast, by the multiplexed HRM assay, 27.17 % (197/725) of subjects were shown to have G6PD mutations. The mutations detected in this study included four single variants, G6PD Mahidol (187/197), G6PD Canton (4/197), G6PD Viangchan (3/197) and G6PD Chinese-5 (1/197), and two double mutations, G6PD Mahidol + Canton (1/197) and G6PD Chinese-4 + Viangchan (1/197). A broad range of G6PD enzyme activities were observed in individuals carrying G6PD Mahidol, especially in females.

**Conclusions:**

The multiplexed HRM-based assay is sensitive and reliable for detecting G6PD mutations. This genotyping assay can facilitate the detection of heterozygotes, which could be useful as a supplementary approach for high-throughput screening of G6PD deficiency in malaria endemic areas before the administration of primaquine and tafenoquine.

## Background

Glucose-6-phosphate dehydrogenase (G6PD) deficiency is an inherited genetic defect and the most common enzymopathy, affecting approximately 500 million people worldwide with more than 200 variants have been identified [1]. G6PD deficiency is prevalent in tropical and subtropical areas where malaria is endemic, including Africa and Southeast Asia [2]. Evidence has suggested that G6PD deficiency confers protection against malaria infection [3–5]. However, this is still controversial because several studies have yielded contradictory results with some claiming that the protective effects of G6PD deficiency were observed in male hemizygotes only, in female heterozygotes only, or in both [6–9]. The major clinical concern associated with G6PD deficiency is haemolysis upon exposure to oxidant drugs, including anti-malarials such as 8-aminoquinolines (primaquine and tafenoquine) [10–13]. Primaquine and tafenoquine are the only medications capable of killing *Plasmodium vivax* and *Plasmodium ovale* at the dormant liver stage (hypnozoite). The World Health Organization (WHO) recommends that G6PD activity be measured before efforts to perform radical treatment of malaria [14].

G6PD deficiency can be diagnosed by either phenotypic or genotypic assay. Phenotypic tests are based on the assessment of G6PD activity, measuring the production of reduced nicotinamide adenine dinucleotide phosphate (NADPH), which can be done quantitatively. The standard quantitative method is spectrophotometry, in which NADPH production is monitored at 340 nm [15]. This method is accurate and reliable, but is laborious, time-consuming, and requires complicated sample preparation and technical skills; as such, it is not commonly used for field-based screening. A colorimetric G6PD assay, based on water-soluble tetrazolium salts (WST-8), was developed as an alternative to the gold standard of spectrophotometry [16]. In this approach, no sample preparation is required and whole blood or dried blood spots can be used to test G6PD activity [17]. The WST-8-based assay can be used as a quantitative method or a qualitative one by the naked eye, offering the possibility of performing mass screening of G6PD deficiency in the context of malaria elimination using primaquine and tafenoquine [16, 18]. Although not the standard method for measuring G6PD activity, the sensitivity of WST-8 for detecting NAD(P)H was found to be five-fold greater than that of the spectrophotometric assay. Moreover, results obtained by measuring dehydrogenase activities in biological samples using WST-8 assay were in parallel with the standard method [19]. For G6PD testing, WST-8 was applied, in 96-well format, to the screening of G6PD deficiency in different populations [20–22]. The sensitivity and specificity of WST-8 for detecting G6PD activity < 30 % were 55 % and 98 %, respectively, compared with the spectrophotometric method [20]. In addition, sensitivity of 72 % and specificity of 98 % were reported for WST-8, in comparison with the standard quantitative G6PD assay (R&D Diagnostics) [21]. This suggests that WST-8 could be a key tool for G6PD testing, but it requires further development before deployment in the field.

G6PD diagnostic tests are currently available, including qualitative tests such as fluorescent spot test (FST) and CareStart™ G6PD rapid diagnostic test, as well as quantitative point-of-care tests such as CareStart™ G6PD biosensor and STANDARD™ G6PD test. Unfortunately, these tests are not widely used for G6PD testing because they are too expensive and can be difficult to interpret [23–25]. Qualitative tests are reliable for identifying G6PD deficiency in hemizygous males and homozygous females, but are unable to identify heterozygous females [26–28]. This is because, in heterozygous females, a wide range of G6PD activities are observed as a result of the random X-chromosome inactivation or lyonization [29]. To date, over 200 G6PD variants have been identified, which form genotypes associated with differences in the degree of deficiency and vulnerability to haemolysis [30]. Moreover, G6PD activities vary among G6PD-deficient individuals carrying the same genotype [31, 32].

G6PD genotyping can be performed using restriction fragment length polymorphism [33, 34], amplification refractory mutation system [35, 36], gold nanoparticles-based assay [37], high resolution melting (HRM) curve analysis [38, 39] and DNA sequencing [40, 41]. Additionally, multiplex genotyping systems are currently available. DiaPlexC™ G6PD Genotyping Kit (Asian type) can detect eight mutations, namely, G6PD Vanua Lava, G6PD Mahidol, G6PD Mediterranean, G6PD Coimbra, G6PD Viangchan, G6PD Union, G6PD Canton, and G6PD Kaiping. Thus, this assay offers high-throughput screening of G6PD mutations by one-step PCR [42]. However, after PCR amplification, an additional gel electrophoresis step is required to check the size of the amplified PCR products, which is impractical for large population screening. The HRM assay is a powerful and reliable tool that has been widely used in the detection of gene mutations [43–45]. Previously, HRM assays were applied to detect G6PD mutations in different population groups [38, 46–48]. However, previous HRM assays could detect only one or two mutations at a time. Although a multiplexed system to detect six mutations in four reactions was later described, the assay system and interpretation of results were complex [49].

The prevalence of G6PD deficiency in Thailand ranges between 5 and 18 %, depending on the geographical area [50–54]. More than 20 G6PD variants have been identified in the country, among which the most common is G6PD Viangchan, followed by G6PD Mahidol, G6PD Canton, G6PD Union, G6PD Kaiping, G6PD Gaohe, G6PD Chinese-4, G6PD Chinese-5, G6PD Valladolid, G6PD Coimbra and G6PD Aures. Along the Thai–Myanmar border, a malaria endemic area, prevalence of G6PD deficiency of 9–18 % was reported in males [26]. Moreover, a rate of G6PD deficiency of 7.4 % was reported from the screening of 1,340 newborns [27]. G6PD Mahidol was shown to be the most common variant in this population, accounting for 88 % of all variants, followed by G6PD Chinese-4, G6PD Viangchan, and G6PD Mediterranean. Generally, to avoid the risk of haemolysis upon malaria treatment, G6PD testing is recommended before the administration of primaquine and tafenoquine. The aim of this study was to develop a molecular diagnostic test to enable an accurate, reliable and high-throughput platform for detecting G6PD mutations, which can be used as a supplement to the screening of G6PD deficiency, especially in heterozygous females. To validate the method, 70 G6PD-deficient and 28 non-deficient samples were tested and the results were compared with the findings obtained by direct DNA sequencing. The potential utility of the developed HRM test for the detection of G6PD variants in a study area in Thailand was then examined. The correlation between genotype and the phenotype of enzyme activity (as determined using WST-8) was also analysed.

## Methods

### Blood samples

Blood samples were collected in ethylenediaminetetraacetic acid (EDTA) tubes and transported to the laboratory under storage at 4 °C. Thereafter, samples were stored at −20 °C until use, for approximately 1−3 months. Under these conditions, the integrity of samples for phenotypic analysis was maintained as it was recently reported that blood samples were stable for up to 7–12 months when stored in EDTA tubes at − 20 °C [55].

For the validation of HRM assays, 70 G6PD-deficient and 28 non-deficient blood samples were collected from healthy Thai volunteers at the Faculty of Medicine Ramathibodi Hospital. All samples were spectrophotometrically tested for G6PD activity and genotyped by DNA sequencing. Ethical approval for the study was provided by the Committee on Human Rights Related to Research Involving Human Subjects, Faculty of Medicine Ramathibodi Hospital, Mahidol University, Bangkok, Thailand (approval number MURA 2018/252).

For the screening of G6PD deficiency, 725 blood samples (from 368 males and 357 females) were collected in EDTA tubes from residents living along the Thai–Myanmar border, a malaria endemic area, namely, in Tha Song Yang District, Tak Province, Thailand. Ethical approval for the study was provided by the Human Ethics Committee, Faculty of Tropical Medicine, Mahidol University (approval number MUTM 2019-016-01).

### Phenotypic screening of G6PD deficiency using WST-8 assay

WST-8 is not a standard method for measuring G6PD activity. However, this assay was used for phenotypic screening in this study because its performance was found to be indistinguishable from that of the spectrophotometric method involving measurement of the absorbance of NAD(P)H at 340 nm [19]. The method showed high accuracy with % relative error of 0.7–0.25. For precision, % coefficient of variation for within-run and between-run of the WST-8 method ranged between 0.6 and 4.5. WST-8 also exhibited excellent reproducibility with Z′ values of 0.90–0.99. Although WST-8 provides advantages regarding the diagnosis of G6PD deficiency, this method will require further development before being deployed in a clinical context [20].

Reaction mixtures of 100 µl, consisting of 20 mM Tris-HCl pH 8.0, 10 mM MgCl_2_, 500 µM glucose-6-phosphate (G6P), 100 µM NADP^+^, and 100 µM WST-8 (Sigma-Aldrich, Darmstadt, Germany), were mixed with a blood sample of 2 µl in a 96-well plate. The absorbance was measured at 450 nm with a reference at 650 nm using a microplate reader (Sunrise; Tecan, Männedorf, Switzerland). The absorbance at 450 nm of a reaction mixture set up in the absence of G6P substrate was used for background subtraction. The G6PD activity was calculated using an NADPH calibration curve. Haemoglobin concentration was measured using Drabkin’s reagent (Sigma-Aldrich). G6PD activity was reported as units (U) per gram of haemoglobin (gHb). Experiments were performed in triplicate.

### DNA extraction

DNA extraction was performed using the QIAsymphony DNA Mini Kit (QIAGEN, Hilden, Germany), in accordance with the manufacturer’s instructions. Blood samples of 100 µl were extracted and eluted into a final volume of 50 µl. DNA concentration was measured using a NanoDrop 2000 spectrophotometer (Thermo Fisher Scientific, Waltham, MA, USA).

### Primer design

**Fig. 1:**
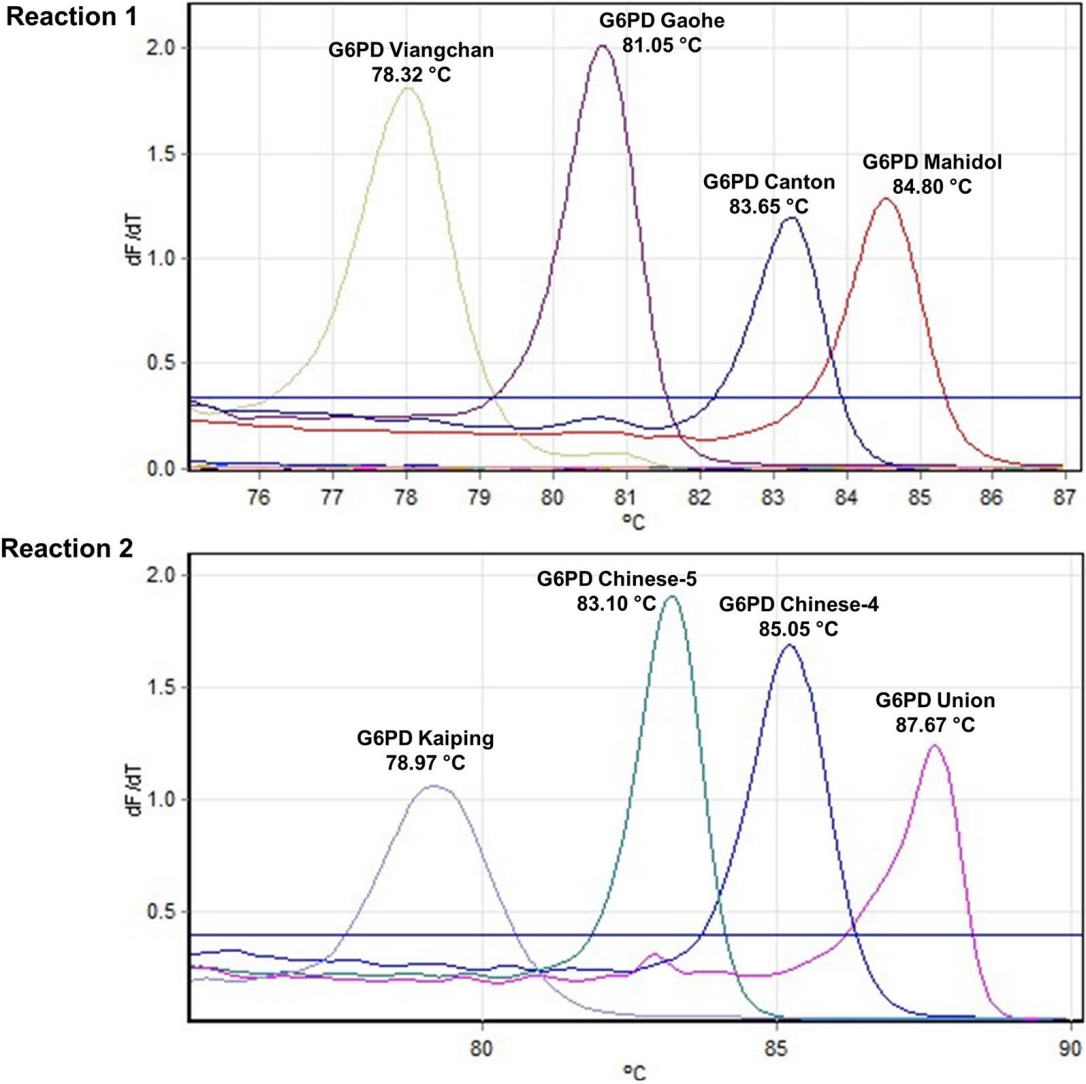
Identification of G6PD mutations by the multiplexed HRM assay. The assay is based on base complementarity between primers and the DNA template. Mutant samples produce a peak at the corresponding T_m_, while WT samples do not produce PCR products, giving a flat line

Primers were designed to detect eight common G6PD mutations in the Thai population: G6PD Gaohe (A95G), G6PD Chinese-4 (G392T), G6PD Mahidol (G487A), G6PD Viangchan (G871A), G6PD Chinese-5 (C1024T), G6PD Union (C1360T), G6PD Canton (G1376T) and G6PD Kaiping (G1388A; Table 1). The primers were designed to detect the mutations by generating PCR products with distinctive melting temperatures (T_m_; Fig. 1).

**Table 1:** HRM primers used in this study

Reaction system	Primer name	G6PD variant	Primer sequence (from 5’ to 3’)	Primer concentration (nM)	Amplicon size (bp)	T_m_ of PCR product (°C)
1	A95G_F	Gaohe	TTCCATCAGTCGGATACACG	600	100	81.05
	A95G_R	(His32Arg)	AGGCATGGAGCAGGCACTTC	600		
	G487A_F	Mahidol	TCCGGGCTCCCAGCAGAA	400	87	84.80
	G487A_R	(Gly163Ser)	GGTTGGACAGCCGGTCA	400		
	G871A_F	Viangchan	GGCTTTCTCTCAGGTCAAGA	400	66	78.32
	G871A_R	(Val291Met)	CCCAGGACCACATTGTTGGC	400		
	G1376T_F	Canton	CCTCAGCGACGAGCTCCT	600	99	83.65
	G1376T_R	(Arg459Leu)	CTGCCATAAATATAGGGGATGG	600		
2	G392T_F	Chinese-4	CATGAATGCCCTCCACCTGGT	200	87	85.05
	G392T_R	(Gly131Val)	TTCTTGGTGACGGCCTCGTA	200		
	C1024T_F	Chinese-5	CACTTTTGCAGCCGTCGTCT	400	99	83.10
	C1024T_R	(Leu342Phe)	CACACAGGGCATGCCCAGTT	400		
	C1360T_F	Union	GAGCCAGATGCACTTCGTGT	200	127	87.67
	C1360T_R	(Arg454Cys)	GAGGGGACATAGTATGGCTT	200		
	G1388A_F	Kaiping	GCTCCGTGAGGCCTGGCA	400	57	78.97
	G1388A_R	(Arg463His)	TTCTCCAGCTCAATCTGGTGC	400		

### PCR amplification and melting curve analysis

Assay conditions, including primer concentrations, assay protocol, and detection conditions, were optimized to maximize the sensitivity and specificity of the assay and to minimize the cross-reactivity. Multiplexed HRM assay was performed in a total volume of 12.5 µl, containing 6.25 µl of 2× HRM Type-It mix (QIAGEN), various concentrations of each primer (Table 1), molecular-grade water and 2.5 µl of the gDNA template (3–10 ng/µl). PCR amplification and melting curve analysis were performed using the Rotor-Gene Q (QIAGEN) with the following conditions: 1 cycle of 95 °C for 5 min, and then 30 cycles of 95 °C for 10 s, 63 °C for 30 s, and 72 °C for 10 s. Subsequently, HRM analysis was performed by melting from 75 to 90 °C, reading at every 0.1 °C step with 2 s of stabilization. Positive (gDNA with known mutations, confirmed by DNA sequencing) and negative controls (gDNA of G6PD wild-type (WT), confirmed by DNA sequencing) were included in every run. Data analysis was carried out using the Rotor-Gene Q software. Experiments were performed in triplicate.

#### PCR amplification and DNA sequencing

To validate the HRM results, PCR and sequencing primers were designed, as shown in Table 2. For DNA amplification, extracted gDNA was used as a template. The *g6pd* gene was amplified using four primer sets (Exon2F−Exon2R, Exon3F−Exon5R, Exon6F−Exon8R, and Exon9F−Exon13R), which cover all 13 exons. The PCR reaction was set up in a final volume of 50 µl, containing 1⋅ Taq Buffer with (NH_4_)_2_SO_4_, 2.5 mM MgCl_2_, 200 µM of each dNTP, 0.25 µM of each primer, 50 ng gDNA and 1.25 U of Taq DNA polymerase (Thermo Fisher Scientific). The thermal cycling profile was as follows: initial denaturation at 95 °C for 3 min; 35 cycles of denaturation at 95 °C for 30 s, annealing for 30 s, and extension at 72 °C for 1 min; followed by final extension at 72 °C for 10 min. The annealing temperature was 60 °C for the primers Exon2F−Exon2R, Exon3F−Exon5R, and Exon6F−Exon8R, and 65 °C for Exon9F−Exon13R. PCR products were subjected to gel purification and sequenced (Bio Basic, Ontario, Canada).

**Table 2:** Sequencing primers used in this study

Primer name	Primer sequence (from 5’ to 3’)
Exon2F	GGGCAATCAGGTGTCACC
Exon2R	GGCTTTTAAGATTGGGGCCT
Exon3F	AGACATGCTTGTGGCCCAGTA
Exon5F	GGACACTGACTTCTGAGGGCA
Exon5R	AAGGGAGGGCAACGGCAA
Exon6F	CACGGGGGCGAGGAGGTT
Exon8F	CGGTTTTATGATTCAGTGATA
Exon8R	AGGGCATGCTCCTGGGGA
Exon9F	GTGAGCAGAGCCAAGCAG
Exon11F	CAGATACAAGGTGCCCTACAG
Exon13R	TGGCGGGGGTGGAGGTGG

### Statistical analysis

Data are presented as mean ± SD. Statistical analyses and plotting of graphs were performed using GraphPad Prism (GraphPad Software, La Jolla, CA, USA). To assess the performance of multiplexed HRM in the detection of G6PD mutations, the numbers of true positives, true negatives, false positives, and false negatives were determined. The following parameters were calculated: sensitivity = [true positives/(true positives + false negatives)] ⋅100; specificity = [true negatives/(true negatives + false positives)] ⋅100; positive predictive value = [true positives/(true positives + false positives)] ⋅100; and negative predictive value = [true negatives/(true positives + false negatives)] ⋅100.

## Results

### Development and validation of 4-plex HRM assay

A multiplexed HRM assay was developed to detect eight G6PD variants that are common in Thailand in two reactions (Fig. 1). By using a specific primer pair for each mutation, reaction 1 simultaneously detects four mutations [G6PD Gaohe (A95G), G6PD Mahidol (G487A), G6PD Viangchan (G871A), and G6PD Canton (G1376T)]. Reaction 2 concurrently detects another four mutations [G6PD Chinese-4 (G392T), G6PD Chinese-5 (C1024T), G6PD Union (C1360T), and G6PD Kaiping (G1388A)]. The assay is based on a single fluorescent dye, EvaGreen, without the need for a quenching probe. The primers were designed to detect the mutations by generating PCR products with distinctive melting temperatures, T_m_. In contrast, no amplification occurred in WT samples. A peak at the corresponding T_m_ reveals the genotype of each sample. The gDNA of known G6PD mutations was used as positive controls. Overall, 70 G6PD-deficient samples and 28 non-deficient samples were used to evaluate the performance of the developed 4-plex HRM assay, while direct DNA sequencing was used as a reference test (Table 3).

**Table 3:** G6PD mutations of 70-deficient samples detected by 4-plex HRM and direct DNA sequencing

Mutation	HRM assay	DNA sequencing
Gaohe (A95G)	4/70	4/70
Chinese-4 (G392T)	3/70	3/70
Mahidol (G487A)	5/70	5/70
Viangchan (G871A)	28/70	28/70
Chinese-5 (C1024T)	1/70	1/70
Canton (G1376T)	14/70	14/70
Kaiping (G1388A)	13/70	13/70
Mahidol + Canton (G487A + G1376T)	1/70	1/70
Gaohe + Kaiping (A95G + G1388A)	1/70	1/70

In comparison to direct DNA sequencing, the 4-plex HRM assay was 100 % sensitive [95 % confidence interval (CI): 94.87–100 %] and 100 % specific (95 % CI: 87.66–100 %), with no cross-reactivity for the detection of G6PD mutations (Table 4). Additionally, the multiplexed HRM assay could correctly identify the double mutations (G6PD Mahidol + Canton and G6PD Gaohe + Kaiping). This indicates that the developed method is reliable for detecting G6PD mutations.

**Table 4:** Performance of the HRM assay for the identification of G6PD mutations

Parameter	HRM assay
True positive	70/70
True negative	28/28
False positive	0/28
False negative	0/70
Sensitivity	100 %
Specificity	100 %
Positive predictive value	100 %
Negative predictive value	100 %

### Phenotypic screening of G6PD deficiency by WST-8 assay

**Fig. 2:**
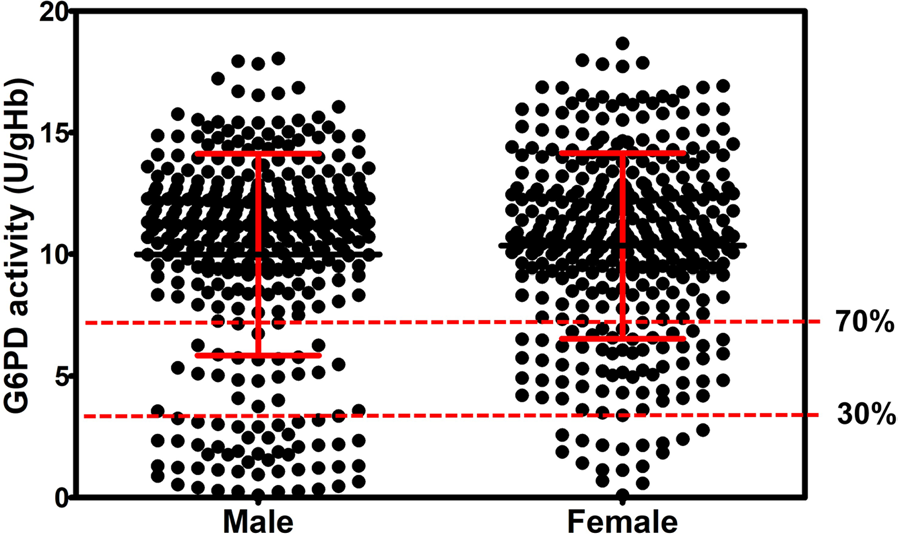
G6PD activity of 725 individuals (368 males and 357 females) measured by the WST-8 method. The adjusted male median (AMM) value was determined to be 10.31 ± 3.81 U/gHb and defined as 100 % G6PD activity. Dotted horizontal lines indicate G6PD activity at 30 and 70 % of the AMM

The prevalence of G6PD deficiency in people living in a malaria endemic area in Thailand, namely, Tha Song Yang District, Tak Province, was determined by G6PD activity assay (WST-8). Figure 2 indicates the G6PD enzyme activity of 725 samples measured by WST-8. The average G6PD activity in males and females was 9.99 ± 4.14 and 10.35 ± 3.81 U/gHb, respectively. The adjusted male median (AMM) value was determined (10.31 ± 3.81 U/gHb) and defined as 100 % G6PD activity [56]. The WHO defined G6PD activity of less than 30 % as deficient and G6PD activity ranging between 30 and 80 % as intermediate [57].

**Fig. 3:**
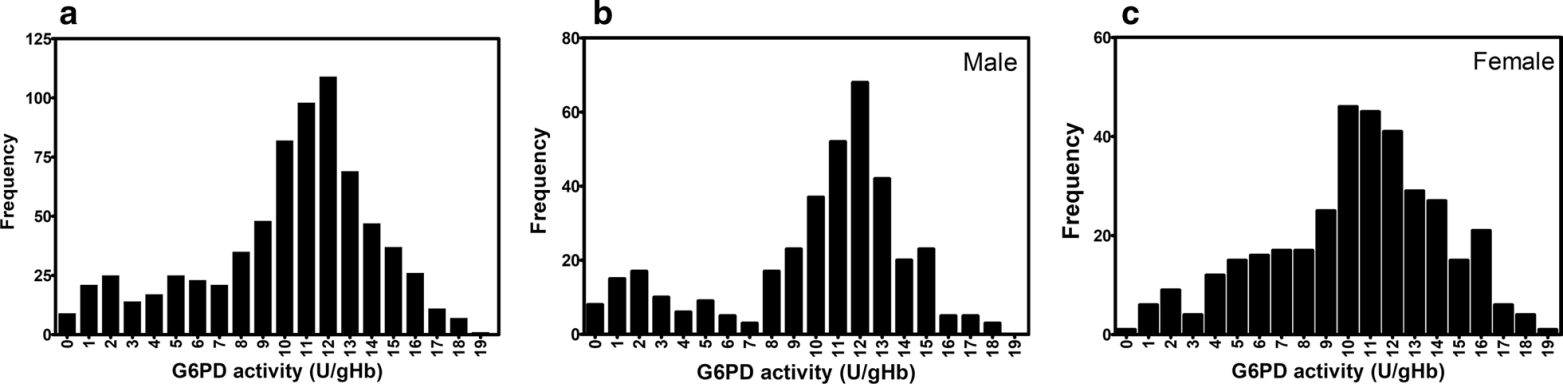
Frequency distribution of G6PD activity. **a** G6PD activity for all 725 samples, showing the majority of samples in the range between 7 and 16 U/gHb. The average G6PD activity of the 725 samples was 10.19 ± 3.96 U/gHb. G6PD activity for (**b**) 368 males and (**c**) 357 females. The average G6PD activity in males and females was 9.99 ± 4.14 and 10.35 ± 3.81 U/gHb, respectively

Nonetheless, G6PD activity of 70 % was used as a threshold for tafenoquine prescription [58, 59]. In this study, G6PD activity levels of less than 30 % and 30–70 % of the AMM were thus considered as deficient and intermediate, respectively. Subjects with G6PD activity over 70 % of the AMM were defined as normal. Based on the WST-8 assay, the prevalence of G6PD deficiency in the studied population was 20.55 % (149/725; Table 5). Prevalence rates of G6PD deficiency of 20.11 % (74/368) and 21.01 % (75/357) were observed in males and females, respectively. In addition, average G6PD activity of deficient males and females was 1.59 ± 0.89 and 1.69 ± 0.77 U/gHb, respectively. Intermediate G6PD activity (30–70 %) was found in 7.34 % (27/368) of males and 16.25 % (58/357) of females. Average G6PD activity of non-deficient (> 70 %) cases was 11.78 ± 2.11 U/gHb in males and 11.89 ± 2.49 U/gHb in females. The frequency distribution of G6PD activity of the 725 individuals measured by WST-8 is shown in Fig. 3a. The majority of the enzyme activities were distributed between 7 and 16 U/gHb. The frequency distribution of G6PD activity by sex is illustrated in Fig. 3b, c. A broader distribution of G6PD activities was seen in females than in males.

**Table 5:** Prevalence of G6PD deficiency determined by WST-8 enzyme activity assay

G6PD status	Male, N (%)	Female, N (%)	Total, N (%)
Deficient (< 30 %)	47 (12.77 %)	17 (4.76 %)	64 (8.83 %)
Intermediate (30–70 %)	27 (7.34 %)	58 (16.25 %)	85 (11.72 %)
Normal (> 70 %)	294 (79.89 %)	282 (78.99 %)	576 (79.45 %)
Total	368 (100 %)	357 (100 %)	725 (100 %)

### **Genotypic screening of G6PD deficiency using the multiplexed HRM assay**

**Fig. 4:**
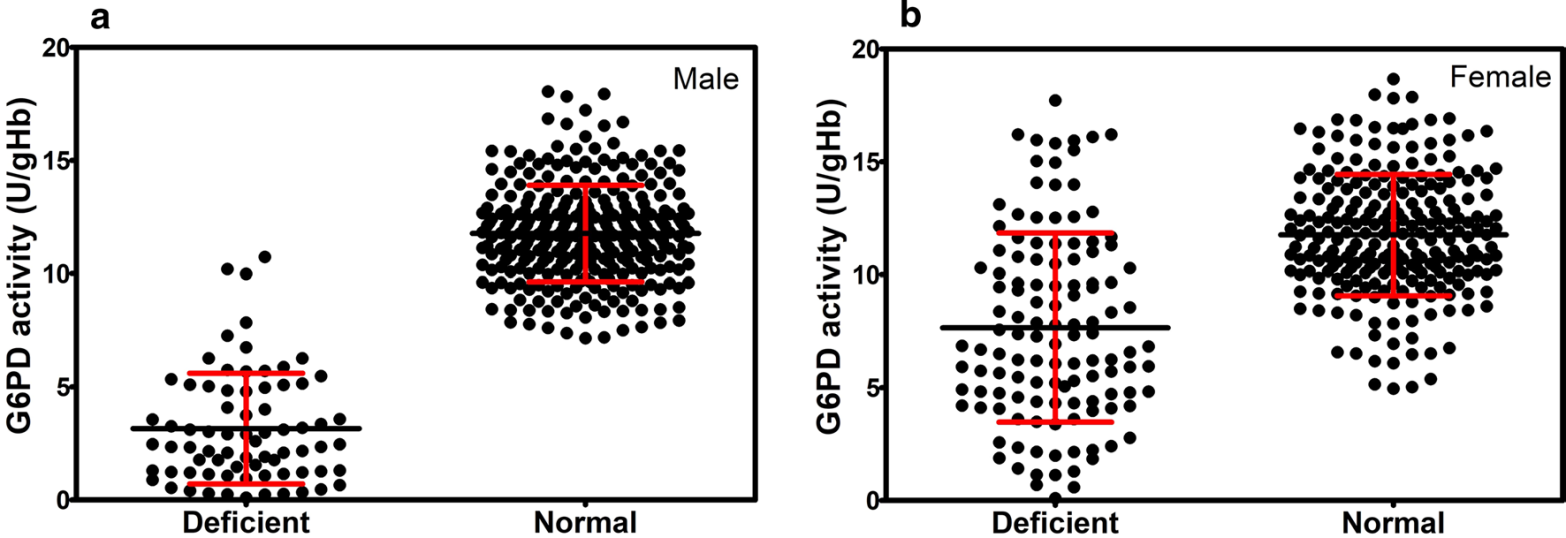
G6PD activity of deficient and normal samples identified by HRM assay. G6PD activity in **a** male and **b** female subjects. The average G6PD activity of deficient males and females was 3.16 ± 2.45 and 7.66 ± 4.19 U/gHb, respectively. The average G6PD activity of normal males and females was 11.77 ± 2.13 and 11.76 ± 2.68 U/gHb, respectively

**Fig. 5:**
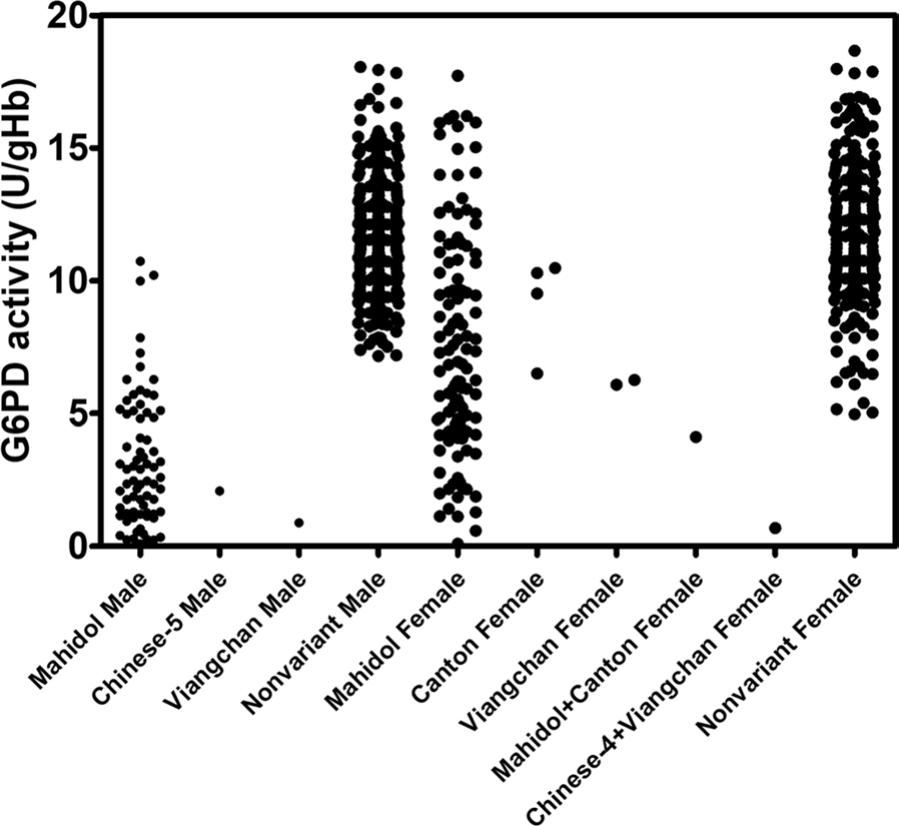
Distribution of G6PD activity by mutation type. Males carrying G6PD Mahidol showed G6PD enzyme activity ranging from 0.10 to 10.73 U/gHb. Females carrying G6PD Mahidol showed a wider range of G6PD enzyme activities (0.10–17.72 U/gHb). Females with G6PD Canton exhibited G6PD activity between 6.50 and 10.48 U/gHb. Females with G6PD Viangchan showed G6PD activity of 6.07–6.25 U/gHb. Normal males showed G6PD activity ranging from 7.16 to 18.05 U/gHb and normal females showed that between 4.96 and 18.67 U/gHb

The developed 4-plex HRM assay was applied to screen for G6PD mutations in 725 blood samples. This assay identified 197 of the 725 (27.17 %) individuals as possessing at least one mutation with an adverse effect on function (Table 6). The prevalence of subjects carrying at least one G6PD mutation was 20.11 % (74/368) in males and 34.45 % (123/357) in females. The most common G6PD mutation detected in the studied population was G6PD Mahidol, accounting for 94.92 % of the total (n = 187; 72 in males and 115 in females). Other single mutations observed in the study included G6PD Canton (2.03 %; 4 in females), G6PD Viangchan (1.52 %; 1 in a male and 2 in females), and G6PD Chinese-5 (0.51 %; 1 in a male). The HRM assay could also detect the double mutant variants, which were G6PD Mahidol + Canton (0.51 %; 1 in a female) and G6PD Chinese-4 + Viangchan (0.51 %; 1 in a female). Figure 4 shows the G6PD activity of deficient and normal samples identified by HRM for males and females. G6PD enzyme activity of deficient subjects, especially in females, spanned from the deficient region (< 30 %) to the normal region (> 70 %). A large distribution of G6PD enzyme activities in females is caused by genetic mosaicism as a result of X-inactivation. The distribution of G6PD activity by mutation type is illustrated in Fig. 5. Non-variant individuals are also included in this plot. Variation of G6PD activities among the different mutations was observed. Moreover, compared with the G6PD enzyme activity in males with the same mutation, that in females was greater. Enzyme activity of 0.89 and 6.16 U/gHb was observed for G6PD Viangchan in males and females, respectively. Interestingly, G6PD Mahidol, a Class III variant with mild deficiency, which was the most prevalent variant in the studied population, exhibited a wide range of G6PD activities, in both males (range: 0.10–10.73 U/gHb, mean: 3.20 ± 2.46 U/gHb) and females (range: 0.10–17.72 U/gHb, mean: 7.72 ± 4.24 U/gHb). Notably, G6PD enzyme activity in the double mutant variants (G6PD Mahidol + Canton and G6PD Chinese-4 + Viangchan) was significantly decreased compared with that of the single mutants.

**Table 6:** Observed ranges of enzyme activity and G6PD genotypes identified by HRM assay

Gender	Variant	N	G6PD activity (U/gHb)	Nucleotide change	Amino acid change	WHO Classification
Male	Mahidol	72	0.10-10.73	G487A	Gly163Ser	III
(n = 368)	Chinese-5 Viangchan	1 1	2.10 0.89	C1024T G871A	Leu342Phe Val291Met	II II
	Non-variant	294	7.16–18.05	-	-	Normal
Female	Mahidol	115	0.10-17.72	G487A	Gly163Ser	III
(n = 357)	Canton	4	6.50-10.48	G1376T	Arg249Leu	II
	Viangchan	2	6.07–6.25	G871A	Val291Met	II
	Mahidol + Canton	1	4.12	G487A + G1376T	Gly163Ser + Arg249Leu	II/III
	Chinese-4 + Viangchan	1	0.69	G392T + G871A	Gly131Val + Val291Met	II
	Non-variant	234	4.96–18.67	-	-	Normal

## Discussion

HRM assays have been widely used to detect gene mutations [43–45]. However, for G6PD genotyping, most of the developed HRM assays are singleplex or duplex, which can only detect one or two mutations simultaneously [38, 46, 47]. To enable the detection of multiple mutations, more than one fluorescent dye must be included in the reaction mixture, which usually makes the reaction more expensive [39, 60, 61].

As reported in this paper, a 4-plex HRM assay for the detection of G6PD mutations common in Thailand and elsewhere in Asia was developed, using a single dye with a run time of 80 min. Evaluation of the 4-plex HRM assay using 70 G6PD-deficient samples indicated that it was accurate and reliable for detecting G6PD mutations (with specificity and sensitivity of 100 %) compared with DNA sequencing. Among the 70 deficient assay validation samples, G6PD Viangchan was the most prevalent variant, followed by G6PD Canton and G6PD Kaiping. This is in accordance with previous reports showing that G6PD Viangchan was the most common variant in Thais [28, 51]. Two double mutations, G6PD Mahidol + Canton and G6PD Gaohe + Kaiping, were also identified in the studied population. G6PD Mahidol + Canton was first identified in people living along the Thai–Myanmar border where Karen and Burman are the major population groups [62].

After validation, the multiplexed HRM assay was applied to screen G6PD mutations of 725 people living in a malaria endemic area along the Thai–Myanmar border. The prevalence of G6PD deficiency in this population was also determined by phenotypic enzyme activity assay using WST-8. Considering a 30 % activity cut-off, the overall prevalence of G6PD deficiency was 8.83 % by WST-8 assay. If the upper limit of a 70 % cut-off was considered, the overall prevalence increased to 20.55 %. By sex, at the 70 % cut-off, the prevalence of G6PD deficiency was 20.11 % in males and 21.01 % in females. In contrast, by the multiplexed HRM assay, the frequency of G6PD mutations was 20.11 and 34.45 % in males and females, respectively. Thus, the prevalence of G6PD deficiency in males is equivalent between these two assays, but in females, genetic analysis using HRM indicated a high frequency of *g6pd* gene mutations (34.45 %), that is notably greater than the prevalence of G6PD deficiency measured by WST-8 (21.01 %). The multiplexed HRM assay identified four single mutants (95.11 % G6PD Mahidol, 2.03 % G6PD Canton, 1.52 % G6PD Viangchan, and 0.51 % G6PD Chinese-5) and two double mutants (0.51 % G6PD Mahidol + Canton and 0.51 % G6PD Chinese-4 + Viangchan) in the studied population. In good agreement with previous reports, G6PD Mahidol was the most common variant in the Karen population [26, 62]. The double mutant G6PD Chinese-4 + Viangchan was identified here for the first time.

A broad range of G6PD activities were observed among the different genotypes. G6PD Mahidol showed ranges of enzyme activity of 0.10–10.73 and 0.10–17.72 U/gHb in males and females, respectively. For G6PD Canton, the range of enzyme activity was 6.50–10.48 U/gHb in females. A wider distribution of enzyme activities was observed in females carrying G6PD Mahidol than in males. Additionally, 5 males and 58 females carrying G6PD Mahidol and 3 females carrying G6PD Canton showed G6PD activity even greater than the 70 % activity cut-off (7 U/gHb). Similar findings were previously described in genotype–phenotype association studies [31, 32]. This is mainly attributable to the fact that the degree of deficiency and vulnerability to haemolysis vary substantially among the different genotypes. Furthermore, because G6PD deficiency is an X-linked genetic defect, males exhibit hemizygous deficiency while females can exhibit either homozygous or heterozygous deficiency. In heterozygous females, a wide range of G6PD activities can be observed, in which the activity of the normal erythrocyte population may compensate for the lost activity of the deficient erythrocyte population. In addition, abnormally high G6PD activity observed in persons carrying G6PD mutations could also be attributable to the following factors. The first is the limited performance quality of the WST-8 used in this study. The second is elevated reticulocyte count in blood samples. Young red blood cells usually exhibit greater G6PD enzyme activity than aged red blood cells. The last is the presence of leukocytes in tested samples (in this study, G6PD activity was measured from whole blood samples). This is because leukocytes retain their nucleus and therefore can continuously synthesize the G6PD enzyme.

G6PD-deficient individuals have increased susceptibility to haemolysis upon exposure to oxidative agents, including primaquine and tafenoquine which are the only medications effective for the radical treatment of infection by *P. vivax* and *P. ovale*. To ensure successful and safe treatment, a single dose of 300 mg of tafenoquine or 0.5 mg/kg/day primaquine for 14 days should be prescribed only in patients with at least 70 % G6PD activity [63]. The effect of the primaquine dose on haemolysis was reported to differ between G6PD normal and G6PD Mahidol heterozygous individuals [64]. These heterozygous females were identified as normal by FST assay while the quantitative assay revealed G6PD activity of 62 and 99 %. Tafenoquine was also reported to cause dose-dependent haemolysis in G6PD Mahidol heterozygous individuals with enzyme activity of 61–80 % [13]. As such, drug-induced haemolysis associated with G6PD deficiency depends on two major factors: the first is the level of G6PD activity, which is determined by G6PD genotype, and the second is the exposure to oxidative stress, namely, metabolites of antimalarial drugs (8-aminoquinolines). Therefore, the drug dose and the ability to metabolize the parent compounds also contribute to the severity of haemolysis in malaria treatment.

Currently, the cut-off of 70 % AMM is widely accepted as an appropriate threshold to determine whether or not to administer tafenoquine [58, 59, 63]. However, the cut-off value should be carefully defined and tested in each population group. Based on the obtained results, to enable accurate determination of the prevalence of G6PD deficiency using WST-8, different cut-off values are required in males and females. The upper limit of 70 % of AMM is recommended for males. However, in heterozygous females, neither the lower (30 %) nor the upper (70 %) limit is reliable for screening G6PD deficiency. It should be noted that the results reported here are based on the WST-8 assay, which is an alternative to the standard method for measuring G6PD activity. Upon using other G6PD tests, the results might be different. For male populations, phenotype tests are useful for G6PD deficiency screening and to enable safe treatment. In contrast, in heterozygous females in whom a wide range of G6PD activities are observed, phenotypic enzyme assay alone might be insufficient to identify G6PD deficiency. Hence, alternative approaches such as genetic analysis could be useful for determining whether drugs should be administered in populations suspected of having G6PD deficiency. The multiplexed HRM assay developed here could be useful for identifying G6PD variants in Thai populations. Although other multiplex systems for genetic analysis are currently available, they might be unsuitable for large population screening. The G6PD gene chip kit can detect 13–14 mutations common in Chinese populations, but must be combined with a hybridization kit, which is time-consuming [32, 65]. The DiaPlexC™ (Asian type) can simultaneously detect eight mutations, but requires an additional gel electrophoresis step to check the amplified products [42]. Additionally, the kit might not be applicable for deployment in regions where populations carry other G6PD mutations (e.g., G6PD Gaohe, G6PD Chinese-4, and G6PD Chinese-5), for which the kit cannot test.

It should be mentioned that, in this study, no mutation was detected in 12 females who were considered likely to be G6PD-deficient because the observed G6PD activity was lower than 7 U/gHb. This might have been because of the limited performance quality (sensitivity of 55–72 %) of the WST-8 assay used in this study [20, 21]. Alternatively, these subjects might carry G6PD mutations for which the multiplexed HRM assay cannot test. DNA sequencing of the whole *g6pd* gene might be required to detect mutations in such individuals. Additionally, to enable mutational screening in more diverse population groups, the assay should be expanded to include other mutations, such as G6PD Mediterranean, G6PD Valladolid, G6PD Coimbra, and G6PD Aures [26]. It should also be noted that the multiplexed HRM assays developed here are not able to identify zygosity of the samples and, thus, require further development before being deployed. Nevertheless, the HRM assays could be of great use for analysing G6PD mutations in supplement to phenotypic G6PD screening in heterozygous females as well as in populations suspected of having G6PD deficiency. Primarily, G6PD genotyping is being done in G6PD-deficient individuals. However, more data on the genotype–phenotype association of G6PD deficiency in diverse population groups should be obtained, which requires a high-throughput screening platform.

## Conclusions

A multiplexed HRM assay for the detection of eight common G6PD mutations in Thailand was developed. The performance of the assay was excellent, with 100 % specificity and 100 % sensitivity. The prevalence of G6PD mutations in 725 people living in a malaria endemic area along the Thai–Myanmar border was determined to be 27.17 % by HRM, which is greater than the prevalence of G6PD deficiency determined by the WST-8 phenotypic assay (20.55 %). Performing a phenotypic assay alone might thus be inadequate and the result might not be an accurate predictor of G6PD deficiency, especially in heterozygous females. As an option to overcome this problem, the multiplexed HRM assay is rapid, accurate and reliable for detecting G6PD mutations, enabling high-throughput screening. This assay could be useful as a supplementary approach for high-throughput screening of G6PD deficiency before the administration of 8-aminoquinolones in malaria endemic areas.

## Data Availability

All data analysed during the study are included in this published article.

## Abbreviations

AMM: Adjusted male median
CI: Confidence interval
FST: Fluorescent spot test
G6P: Glucose-6-phosphate
G6PD: Glucose-6-phosphate dehydrogenase
Hb: Haemoglobin
HRM: High-resolution melting
NADPH: Reduced nicotinamide adenine dinucleotide phosphate
WHO: World Health Organization
WST: Water-soluble tetrazolium salts
WT: Wild-type

## References

[1] Luzzatto L, Ally M, Notaro R (2020). Glucose-6-phosphate dehydrogenase deficiency. Blood.

[2] Nkhoma ET, Poole C, Vannappagari V, Hall SA, Beutler E (2009). The global prevalence of glucose-6-phosphate dehydrogenase deficiency: a systematic review and meta-analysis. Blood Cells Mol Dis.

[3] Leslie T, Briceno M, Mayan I, Mohammed N, Klinkenberg E, Sibley CH (2010). The impact of phenotypic and genotypic G6PD deficiency on risk of *Plasmodium vivax* infection: a case-control study amongst Afghan refugees in Pakistan. PLoS Med.

[4] Louicharoen C, Patin E, Paul R, Nuchprayoon I, Witoonpanich B, Peerapittayamongkol C (2009). Positively selected G6PD-Mahidol mutation reduces *Plasmodium vivax* density in Southeast Asians. Science.

[5] Awab GR, Aaram F, Jamornthanyawat N, Suwannasin K, Pagornrat W, Watson JA (2021). Protective effect of Mediterranean-type glucose-6-phosphate dehydrogenase deficiency against *Plasmodium vivax* malaria. Elife.

[6] Mbanefo EC, Ahmed AM, Titouna A, Elmaraezy A, Trang NT, Phuoc Long A (2017). Association of glucose-6-phosphate dehydrogenase deficiency and malaria: a systematic review and meta-analysis. Sci Rep.

[7] Bienzle U, Ayeni O, Lucas AO, Luzzatto L (1972). Glucose-6-phosphate dehydrogenase and malaria. Greater resistance of females heterozygous for enzyme deficiency and of males with non-deficient variant. Lancet.

[8] Ruwende C, Khoo SC, Snow RW, Yates SN, Kwiatkowski D, Gupta S (1995). Natural selection of hemi- and heterozygotes for G6PD deficiency in Africa by resistance to severe malaria. Nature.

[9] Uyoga S, Ndila CM, Macharia AW, Nyutu G, Shah S, Peshu N (2015). Glucose-6-phosphate dehydrogenase deficiency and the risk of malaria and other diseases in children in Kenya: a case-control and a cohort study. Lancet Haematol.

[10] Carmona-Fonseca J, Alvarez G, Maestre A (2009). Methemoglobinemia and adverse events in *Plasmodium vivax* malaria patients associated with high doses of primaquine treatment. Am J Trop Med Hyg.

[11] Beutler E (2008). Glucose-6-phosphate dehydrogenase deficiency: a historical perspective. Blood.

[12] Cappellini MD, Fiorelli G (2008). Glucose-6-phosphate dehydrogenase deficiency. Lancet.

[13] Rueangweerayut R, Bancone G, Harrell EJ, Beelen AP, Kongpatanakul S, Mohrle JJ (2017). Hemolytic potential of tafenoquine in female volunteers heterozygous for glucose-6-phosphate dehydrogenase (G6PD) deficiency (G6PD Mahidol variant) versus G6PD-normal volunteers. Am J Trop Med Hyg.

[14] WHO (2016). Testing for G6PD deficiency for safe use of primaquine in radical cure of *P. vivax* and *P. ovale* malaria.

[15] WHO. Standardization of procedures for the study of glucose-6-phosphate dehydrogenase. Report of a WHO Scientific Group. Geneva, World Health Organization. 1967:1–53. 4963040

[16] Tantular IS, Kawamoto F (2003). An improved, simple screening method for detection of glucose-6-phosphate dehydrogenase deficiency. Trop Med Int Health.

[17] Arai M, Kosuge K, Kawamoto F, Matsuoka H (2006). Reactivity of blood samples spotted onto filter papers in the WST-8 method for screening of G6PD deficiency. Acta Med Okayama.

[18] Jalloh A, Tantular IS, Pusarawati S, Kawilarang AP, Kerong H, Lin K (2004). Rapid epidemiologic assessment of glucose-6-phosphate dehydrogenase deficiency in malaria-endemic areas in Southeast Asia using a novel diagnostic kit. Trop Med Int Health.

[19] Chamchoy K, Pakotiprapha D, Pumirat P, Leartsakulpanich U, Boonyuen U (2019). Application of WST-8 based colorimetric NAD(P)H detection for quantitative dehydrogenase assays. BMC Biochem.

[20] Ley B, Alam MS, O’Donnell JJ, Hossain MS, Kibria MG, Jahan N (2017). Comparison of three quantitative methods to estimate G6PD activity in the Chittagong Hill Tracts, Bangladesh. PLoS ONE.

[21] De Niz M, Eziefula AC, Othieno L, Mbabazi E, Nabukeera D, Ssemmondo E (2013). Tools for mass screening of G6PD deficiency: validation of the WST8/1-methoxy-PMS enzymatic assay in Uganda. Malar J.

[22] Kuwahata M, Wijesinghe R, Ho MF, Pelecanos A, Bobogare A, Landry L (2010). Population screening for glucose-6-phosphate dehydrogenase deficiencies in Isabel Province, Solomon Islands, using a modified enzyme assay on filter paper dried bloodspots. Malar J.

[23] Brito MA, Peixoto HM, Almeida AC, Oliveira MR, Romero GA, Moura-Neto JP (2016). Validation of the rapid test Carestart™ G6PD among malaria vivax-infected subjects in the Brazilian Amazon. Rev Soc Bras Med Trop.

[24] Satyagraha AW, Sadhewa A, Elvira R, Elyazar I, Feriandika D, Antonjaya U (2016). Assessment of point-of-care diagnostics for G6PD deficiency in malaria endemic rural eastern Indonesia. PLoS Negl Trop Dis.

[25] Oo NN, Bancone G, Maw LZ, Chowwiwat N, Bansil P, Domingo DJ (2016). Validation of G6PD point-of-care tests among healthy volunteers in Yangon, Myanmar. PLoS ONE.

[26] Bancone G, Chu CS, Somsakchaicharoen R, Chowwiwat N, Parker DM, Charunwatthana P (2014). Characterization of G6PD genotypes and phenotypes on the northwestern Thailand-Myanmar border. PLoS ONE.

[27] Thielemans L, Gornsawun G, Hanboonkunupakarn B, Paw MK, Porn P, Moo PK (2018). Diagnostic performances of the fluorescent spot test for G6PD deficiency in newborns along the Thailand-Myanmar border: a cohort study. Wellcome Open Res.

[28] Nantakomol D, Paul R, Palasuwan A, Day NP, White NJ, Imwong M (2013). Evaluation of the phenotypic test and genetic analysis in the detection of glucose-6-phosphate dehydrogenase deficiency. Malar J.

[29] Harper PS (2011). Mary Lyon and the hypothesis of random X chromosome inactivation. Hum Genet.

[30] Gomez-Manzo S, Marcial-Quino J, Vanoye-Carlo A, Serrano-Posada H, Ortega-Cuellar D, Gonzalez-Valdez A (2016). Glucose-6-phosphate dehydrogenase: update and analysis of new mutations around the world. Int J Mol Sci.

[31] Powers JL, Best DH, Grenache DG (2018). Genotype–phenotype correlations of glucose-6-phosphate–deficient variants throughout an activity distribution. J Appl Lab Med.

[32] He Y, Zhang Y, Chen X, Wang Q, Ling L, Xu Y (2020). Glucose-6-phosphate dehydrogenase deficiency in the Han Chinese population: molecular characterization and genotype-phenotype association throughout an activity distribution. Sci Rep.

[33] D’Urso M, Luzzatto L, Perroni L, Ciccodicola A, Gentile G, Peluso I (1988). An extensive search for RFLP in the human glucose-6-phosphate dehydrogenase locus has revealed a silent mutation in the coding sequence. Am J Hum Genet.

[34] Yoshida A, Takizawa T, Prchal JT (1988). RFLP of the X chromosome-linked glucose-6-phosphate dehydrogenase locus in blacks. Am J Hum Genet.

[35] Maffi D, Pasquino MT, Caprari P, Caforio MP, Cianciulli P, Sorrentino F (2002). Identification of G6PD Mediterranean mutation by amplification refractory mutation system. Clin Chim Acta.

[36] Du CS, Ren X, Chen L, Jiang W, He Y, Yang M (1999). Detection of the most common G6PD gene mutations in Chinese using amplification refractory mutation system. Hum Hered.

[37] Seow N, Lai PS, Yung LY (2014). Gold nanostructures for the multiplex detection of glucose-6-phosphate dehydrogenase gene mutations. Anal Biochem.

[38] Yan JB, Xu HP, Xiong C, Ren ZR, Tian GL, Zeng F (2010). Rapid and reliable detection of glucose-6-phosphate dehydrogenase (G6PD) gene mutations in Han Chinese using high-resolution melting analysis. J Mol Diagn.

[39] Liu Z, Yu C, Li Q, Cai R, Qu Y, Wang W (2020). Chinese newborn screening for the incidence of G6PD deficiency and variant of G6PD gene from 2013 to 2017. Hum Mutat.

[40] Vulliamy TJ, D’Urso M, Battistuzzi G, Estrada M, Foulkes NS, Martini G (1988). Diverse point mutations in the human glucose-6-phosphate dehydrogenase gene cause enzyme deficiency and mild or severe hemolytic anemia. Proc Natl Acad Sci USA.

[41] Poggi V, Town M, Foulkes NS, Luzzatto L (1990). Identification of a single base change in a new human mutant glucose-6-phosphate dehydrogenase gene by polymerase-chain-reaction amplification of the entire coding region from genomic DNA. Biochem J.

[42] Lee J, Kim TI, Kang JM, Jun H, Le HG, Thai TL (2018). Prevalence of glucose-6-phosphate dehydrogenase (G6PD) deficiency among malaria patients in Upper Myanmar. BMC Infect Dis.

[43] Twist GP, Gaedigk R, Leeder JS, Gaedigk A (2013). High-resolution melt analysis to detect sequence variations in highly homologous gene regions: application to CYP2B6. Pharmacogenomics.

[44] Thomas V, Mazard B, Garcia C, Lacan P, Gagnieu MC, Joly P (2013). UGT1A1 (TA)n genotyping in sickle-cell disease: high resolution melting (HRM) curve analysis or direct sequencing, what is the best way?. Clin Chim Acta.

[45] Edwards T, Sasaki S, Williams C, Hobbs G, Feasey NA, Evans K (2018). Speciation of common Gram-negative pathogens using a highly multiplexed high resolution melt curve assay. Sci Rep.

[46] Joly P, Lacan P, Garcia C, Martin C, Francina A (2010). Rapid genotyping of two common G6PD variants, African (A-) and Mediterranean, by high-resolution melting analysis. Clin Biochem.

[47] Pan M, Lin M, Yang L, Wu J, Zhan X, Zhao Y (2013). Glucose-6-phosphate dehydrogenase (G6PD) gene mutations detection by improved high-resolution DNA melting assay. Mol Biol Rep.

[48] Yang H, Wang Q, Zheng L, Zhan XF, Lin M, Lin F (2015). Incidence and molecular characterization of glucose-6-phosphate dehydrogenase deficiency among neonates for newborn screening in Chaozhou, China. Int J Lab Hematol.

[49] Fan Z, Weng X, Huang G, Pan Z, Long Z, Fan Q (2018). STARD-rapid screening for the 6 most common G6PD gene mutations in the Chinese population using the amplification refractory mutation system combined with melting curve analysis. Medicine.

[50] Tanphaichitr VS, Pung-amritt P, Yodthong S, Soongswang J, Mahasandana C, Suvatte V (1995). Glucose-6-phosphate dehydrogenase deficiency in the newborn: its prevalence and relation to neonatal jaundice. Southeast Asian J Trop Med Public Health.

[51] Nuchprayoon I, Sanpavat S, Nuchprayoon S (2002). Glucose-6-phosphate dehydrogenase (G6PD) mutations in Thailand: G6PD Viangchan (871G > A) is the most common deficiency variant in the Thai population. Hum Mutat.

[52] Laosombat V, Sattayasevana B, Janejindamai W, Viprakasit V, Shirakawa T, Nishiyama K (2005). Molecular heterogeneity of glucose-6-phosphate dehydrogenase (G6PD) variants in the south of Thailand and identification of a novel variant (G6PD Songklanagarind). Blood Cells Mol Dis.

[53] Phompradit P, Kuesap J, Chaijaroenkul W, Rueangweerayut R, Hongkaew Y, Yamnuan R (2011). Prevalence and distribution of glucose-6-phosphate dehydrogenase (G6PD) variants in Thai and Burmese populations in malaria endemic areas of Thailand. Malar J.

[54] Tuchinda S, Rucknagel DL, Na-Nakorn S, Wasi P (1968). The Thai variant and the distribution of alleles of 6-phosphogluconate dehydrogenase and the distribution of glucose 6-phosphate dehydrogenase deficiency in Thailand. Biochem Genet.

[55] Chamchoy K, Praoparotai A, Pakparnich P, Sudsumrit S, Swangsri T, Chamnanchanunt S (2021). The integrity and stability of specimens under different storage conditions for glucose-6-phosphate dehydrogenase deficiency screening using WST-8. Acta Trop.

[56] Domingo GJ, Satyagraha AW, Anvikar A, Baird K, Bancone G, Bansil P (2013). G6PD testing in support of treatment and elimination of malaria: recommendations for evaluation of G6PD tests. Malar J.

[57] World Health Organization. Technical specifications series for submission to WHO prequalification–diagnostic assessment: in vitro diagnostic medical devices to identify glucose-6-phosphate dehydrogenase (G6PD) activity. Geneva, World Health Organization, 2016.

[58] Llanos-Cuentas A, Lacerda MVG, Hien TT, Velez ID, Namaik-Larp C, Chu CS (2019). Tafenoquine versus primaquine to prevent relapse of *Plasmodium vivax* malaria. N Engl J Med.

[59] Lacerda MVG, Llanos-Cuentas A, Krudsood S, Lon C, Saunders DL, Mohammed R (2019). Single-dose tafenoquine to prevent relapse of *Plasmodium vivax* malaria. N Engl J Med.

[60] Chen Y, Xiu W, Dong Y, Wang J, Zhao H, Su Y (2018). Mutation of glucose-6-phosphate dehydrogenase deficiency in Chinese Han children in eastern Fujian. Medicine.

[61] Xia Z, Chen P, Tang N, Yan T, Zhou Y, Xiao Q (2016). Rapid detection of G6PD mutations by multicolor melting curve analysis. Mol Genet Metab.

[62] Bancone G, Gilder ME, Chowwiwat N, Gornsawun G, Win E, Cho WW (2017). Prevalences of inherited red blood cell disorders in pregnant women of different ethnicities living along the Thailand-Myanmar border. Wellcome Open Res.

[63] Commons RJ, McCarthy JS, Price RN (2020). Tafenoquine for the radical cure and prevention of malaria: the importance of testing for G6PD deficiency. Med J Aust.

[64] Chu CS, Bancone G, Nosten F, White NJ, Luzzatto L (2018). Primaquine-induced haemolysis in females heterozygous for G6PD deficiency. Malar J.

[65] Hu R, Lin M, Ye J, Zheng BP, Jiang LX, Zhu JJ (2015). Molecular epidemiological investigation of G6PD deficiency by a gene chip among Chinese Hakka of southern Jiangxi province. Int J Clin Exp Pathol.

